# Fast simultaneous detection of *K-RAS *mutations in colorectal cancer

**DOI:** 10.1186/1471-2407-9-179

**Published:** 2009-06-11

**Authors:** Ya-Sian Chang, Kun-Tu Yeh, Tien-Jye Chang, Connie Chai, Hsiu-Chin Lu, Nicholas C Hsu, Jan-Gowth Chang

**Affiliations:** 1 Department of Laboratory Medicine, Kaohsiung Medical University Hospital, Kaohsiung, Taiwan; 2 Department of Medical Research, Kaohsiung Medical University Hospital, Kaohsiung, Taiwan; 3 Department of Veterinary Medicine, National Chung Hsiung University, Taichung, Taiwan; 4 Department of Pathology, Changhua Christian Hospital, Changhua, Taiwan; 5 Institute of Clinical Medicine, Kaohsiung Medical University, Kaohsiung, Taiwan; 6 Center for Excellence in Environmental Medicine, Kaohsiung Medical University, Kaohsiung, Taiwan

## Abstract

**Background:**

*RAS *genes acquire the most common somatic gain-of-function mutations in human cancer, and almost all of these mutations are located at codons 12, 13, 61, and 146.

**Methods:**

We present a method for detecting these *K-RAS *hotspot mutations in 228 cases of colorectal cancer. The protocol is based on the multiplex amplification of exons 2, 3 and 4 in a single tube, followed by primer extension of the PCR products using various sizes of primers to detect base changes at codons 12, 13, 61 and 146. We compared the clinicopathological data of colorectal cancer patients with the *K-RAS *mutation status.

**Results:**

*K-RAS *mutation occurred in 36% (83/228) of our colorectal cancer cases. Univariate analysis revealed a significant association between *K-RAS *mutation at codon 12 of exon 2 and poor 5-year survival (p = 0.023) and lymph node involvement (p = 0.048). Also, *K-RAS *mutation at codon 13 of exon 2 correlates with the size of the tumor (p = 0.03). Multivariate analysis adjusted for tumor size, histologic grade, and lymph node metastasis also indicated *K-RAS *mutations at codon 12 and 13 of exon 2 correlate significantly with overall survival (p = 0.002 and 0.025). No association was observed between codon 61 and 146 and clinicopathological features.

**Conclusion:**

We demonstrated a simple and fast way to identify *K-RAS *mutation.

## Background

The *RAS *genes encode a family of GTPases that act as signal switch molecules for many important cellular processes. Common in human cancers, activating ras mutations cause the deregulation of ras protein activity, which results in the loss of GTPase activity and the gain of oncogenic activity [1-7]. In fact, *RAS *genes are the genes that most commonly show somatic gain-of-function mutations in human cancers [8,9]. There are three cellular *RAS *genes, encoding the K-ras, H-ras and N-ras proteins, and all show activating mutations in human tumors. Of the three *RAS *genes, *K-RAS *is the most frequently mutated, which makes it an ideal target for cancer treatment [9,10]. Meanwhile, *K-RAS *mutation plays an important role of the response rate of anti-EGFR antibodies treatment for patients with metastatic colorectal cancer [11,12].

*RAS *point mutations are highly prevalent in human cancers and mostly occur in codons 12, 13 and 61. These mutations render the ras proteins insensitive to GTP-induced hydrolysis of GTP to GDP and lock them in the activated state [2,8,9]. Mutations in codons 10, 11, 15, 18, 19, and 22 have also been reported, but their biological significance is unclear [13-18]. Several methods have been developed for detecting *K-RAS *mutations [19-26], but these methods do not simultaneously recognize all base changes at codons 12, 13 or 61 of *K-RAS*. Somatic missense mutation at codon 146 were found in three independent studies of colorectal cancers from Hong Kong and the United States, and it was suggested that these mutations may make an equal or greater contribution to colorectal cancer than the codon 61 mutation [27-29]. Direct sequencing of exons 2 and 3 can recognize the base changes in codons 12, 13 and 61. But, codon 146 which is located at exon 4, is too far apart (19,771 base pairs) from exon 2 to be sequenced in a single reaction. In this study, we employed a method to simultaneously detect the base changes in codons 12, 13, 61 and 146 of *K-RAS *in a single tube and establish their clinical significance in colorectal cancer.

## Results

### *K-RAS *mutation detection assay

We designed primers adjacent to codons 12, 13, 61 and 146 of the *K-RAS *gene, as these are the most common mutations in cancer. For mutation analysis of codons 12 and 13, we used different-sized primers (sense strand) to recognize the change of the first base separately, and we designed antisense strand primers to detect the change of the second base of the codon to avoid interference between primers of the same direction. We did not design primers to detect change at the third base of codon 12 or 13 because these mutations do not result in the change of the amino acid. For the analysis of codon 61, we designed three different-sized primers (sense strand) to distinguish changes in the first, second, and third bases separately. The primers were made to be different in size either by adding different lengths of poly(dT) tails to the 5'-end or extending the primer sequence in order to allow for separation based on the differences in size.

First, we analyzed codon 12 mutations using a 20mer sense strand primer to detect the first base changes of codon 12 and a 29mer antisense strand primer to detect the second base changes of codon 12. To the tube with codon 12 primers, we added two more primers to detect the base changes at the first and second bases of codon 13 (41mer sense strand for the first base; 49mer antisense strand for the second base). The results suggested that the primers worked well in spite of the fact that the primers used to detect the first or the second base changes of codons 12 and 13 are of the same direction, suggesting that these primers do not interfere with each other. The extended products were analyzed on the Beckman Coulter automatic sequencer in a 17-minute run, and all the base changes of codons 12 and 13 were detected. These results indicated that the direction of the primer do not play an important role in detecting the base changes at a given codon. We then used different-sized primers of the same direction (sense strand) to recognize the first and the second base changes separately, and all the base changes of codons 12 and 13 were detected. We also used degenerate probes (containing a degenerate base at the last base of the probe to detect the second base change of codon 12 or 13) and they also worked well (data not shown). After this preliminary study using four probes to detect codon 12 and codon 13 mutations, we added five more probes to detect codon 61 and codon 146 mutations. This collection of probes defines our multiplex PCR and primer extension method for codons 12, 13, 61, and 146.

We then used our multiplex PCR and primer extension method to assay 228 cases of colorectal cancer. The results showed that 83 cases have *K-RAS *mutations in the hotspots that we screened. Of the 83 mutant cases, there were 58 cases of codon 12 mutations including 7 cases of GGT→TGT, 3 cases of GGT→AGT, 18 cases of GGT-GTT, 29 cases of GGT→GAT and 1 case of GGT→GCT. There are 21 cases of codon 13 mutations, including 1 case of GGC→CGC and 20 cases of GGC→GAC. There was one example showing a mutation of codon 61 (CAA→CAT), and there were three cases of codon 146 mutations, all of which were of the GCA→ACA mutation. The mutations in all of these cases were heterozygous.

### Sensitivity of the *K-RAS *mutation assay

**Figure 1:**
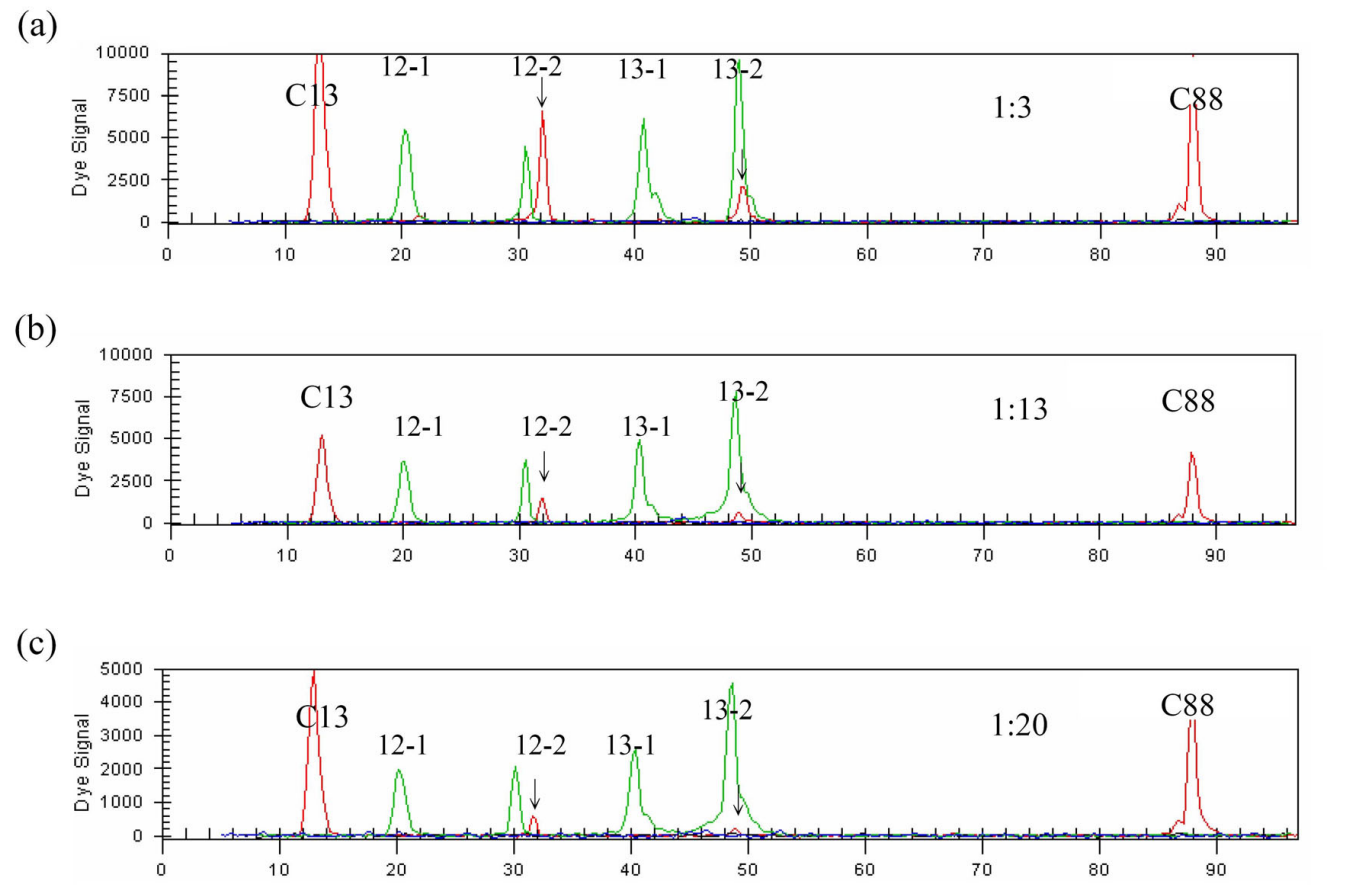
**Determining the sensitivity of multiplex PCR and primer extension analysis of *K-RAS *mutation by serial dilution of the samples**. Upper (a), middle (b) and lower (c) panels are 1: 3, 1: 13 and 1: 20 dilution, respectively.

We next determined the minimal amount of DNA required for reliable mutation detection. We diluted DNA samples harbouring codon 12 and 13 mutations, and analyzed the samples as described above. For each of the mutations, we found that 10 ng of DNA was enough for the multiplex PCR reaction. We subsequently determined the lowest concentration of mutant DNA in a background of normal DNA needed for mutation detection. We mixed tumor DNA (heterozygous for the mutation at codons 12 and 13) with control DNA in different ratios (1:3, 1:13 and 1:20) (Fig. 1). The result showed that this method can detect *K-RAS *mutations in a DNA mixture containing as little as 5% mutant DNA.

### Mutational analysis of *K-RAS *gene in colorectal cancers

We used direct sequencing to analyze the mutations of the *K-RAS *gene in the coding and intron-exon junction regions of exons 2, 3, and 4. As expected, the results showed hotspot *K-RAS *mutation in 83 of the 228 cases. The results of sequencing analysis were identical to the results of multiplex PCR with primer extension analysis. There were no compound heterozygous mutations or homozygous mutations in any of these cases, and the mutational frequencies of codons 12, 13, 61, and 146 were 25.4%, 9.2%, 0.4% and 1.3%, respectively. We did not find any other mutations, such as mutations at codons 10, 11, 15, 18, 19 or 22. We can therefore conclude that our method was very accurate in profiling the mutations in colorectal cancers, and will detect the majority, if not all, of the *K-RAS *mutations commonly seen in cancer.

### Correlation between *K-RAS *mutations and clinicopathological data in colorectal cancers

We compared the clinicopathological data of colorectal cancer patients with the mutational status of *K-RAS *in their cancerous tissues. The univariate analysis revealed that there is a significant association between *K-RAS *mutation at codon 12 of exon 2 and poor 5-year survival. This mutation also serves as a good indicator of lymph node involvement in this disease (Table 1). Furthermore, *K-RAS *mutation at codon 13 of exon 2 correlates with the size of the tumor (>3 cm, p = 0.03). In the multivariate analysis which incorporated independent prognostic factors of tumor size, histologic grade, and lymph node metastasis, we found that *K-RAS *mutations at codon 12 and 13 of exon 2 were both significant with regard to overall survival (p = 0.002 and 0.025) (Table 2). Lymph node involvement also correlates significantly with overall survival (p = 0.001). No association was observed between codon 61 and 146 and clinicopathological features.

**Table 1:** Correlation between clinicopathological features and *K-RAS *mutation of codon 12 1^st ^base and codon 13 2^nd ^base

		mutation of codon 12 1^st ^base				mutation of codon 13 2^nd ^base			
			
		no	yes	total	p-value	no	yes	total	p-value
Tumor size	<= 3 cm	55	5	57	1	56	1	57	0.03
	>3 cm	160	8	168		149	19	168	
									
Grade	Well	4	0	4	0.749	4	0	4	0.416
	Moderate	171	9	180		161	19	180	
	Poor	10	1	11		11	0	11	
									
Lymph node metastasis	-	107	2	109	0.048	101	8	109	0.621
	+	88	8	96		87	9	96	
									
Stage	I, II	76	0	76	0.121	70	6	76	0.782
	III, IV	76	4	80		72	8	80	
									
Survival	<= 5 years	114	9	123	0.023	109	14	123	0.162
	>5 years	104	1	105		99	6	105	

p-value by Chi-square Test or Fisher's Exact Test when appropriated

**Table 2:** Multivariate analysis (Cox regression) of independent prognostic factors in patients with colorectal cancer

		Harzard Ratio	95% CI	p-value
Mutation Codon 12 1st base	-	1.0	1.14 to 4.71	0.002
	+	2.3		
Codon 13 2nd base	-	1.0	1.09 to 3.58	0.025
	+	2.0		
				
Tumor size	<= 3 cm	1.0	0.60 to 1.48	0.803
	>3 cm	0.9		
				
Grade	well	1.0	0.26 to 2.69	0.762
	moderate/poor	0.8		
				
Lymph node metastasis	-	1.0	1.33 to 2.86	0.001
	+	2.0		

## Discussion and Conclusion

*K-RAS *mutations are common in human cancers and play a very important role in various processes of cancer development, including cancer initiation, metastasis, prognosis and response for treatment [1-7,10,11,30-32]. Therefore, mutation detection of *K-RAS *is of clinical importance in cancer studies. In this study, we present a simple assay to detect *K-RAS *mutations in colorectal cancer. Several methods can be used to detect *K-R*AS mutations, including PCR and direct sequencing, PCR-RFLP and direct sequencing, PCR-SSCP and direct sequencing, PCR and probe hybridization, and allele-specific competitive blocker PCR [19-26,33,34]. These methods are laborious because the mutation hotspots in exons 2, 3 and 4 have to be screened separately. Furthermore, the common mutations seen at codons 12, 13, 61 and 146 are not the result of any single nucleotide change; instead, every base at these codons has 3 or 4 types of base changes. It is therefore difficult to use the above-mentioned methods to precisely detect all the base changes without using direct sequencing or hybridization. Although the probe hybridization method requires no direct sequencing, it requires several probes to accurately determine base changes. With our method, the detection of base changes in three key exons of *K-RAS *can be combined into one assay, which allows a sample to be screened for all *K-RAS *mutation hotspots simultaneously. Because the technique is a sequencing-based approach, additional sequencing is not necessary. Meanwhile, the advantage of using primer extension over hybridization with allele-specific oligonucleotide probes to distinguish sequence variants is based on the high accuracy of the nucleotide incorporation reaction catalyzed by a DNA polymerase compared with the caveats of hybridization. These caveats include differences in thermal stability between mismatched and perfectly matched hybrids formed with the allele-specific oligonucleotide probes. Current products of the thermostable enzymes used in primer extension reaction have very low error rates and have increased efficiency and specificity for ddNTPs [35]. These characteristics provide negligible primer misincorporation and excellent discrimination between wild, heterozygous and homozygous genotypes. Additionally, the same reaction conditions can be used to detect any variable nucleotide regardless of the nucleotide sequence flanking the variable site. Another advantage of the primer extension reaction is its multiplexing capability, with several mutations being detected in a single reaction tube. Although multiplex PCR-SSCP- or PCR-ARMS- based methods can also simultaneously detect several mutations, PCR-SSCP require further confirmation by direct sequencing, and using PCR-ARMS to detect all the changes at codons 12, 13, 61 and 146 require more primers than are possible for one reaction tube. Our multiplex primer extension methods can detect more than 40 types of changes at these 4 codons. Compared with direct sequencing, primer extension reaction assays are faster because automated fluorescent capillary electrophoresis of the products requires only 25 minutes in comparison with more than one hour for capillary electrophoresis require for standard sequencing. However, we should point out that our proposed method can only detect *K-RAS *mutations in a DNA mixture containing at least 5% mutant DNA. Moreover, our method can not detect *K-RAS *mutations occurred outside the codons that we targeted although direct sequencing we performed did not find any either. In the past, primer extension-based methods were used for detecting a single base change across several conditions, and they have rarely been used to detect the three base changes at a given codon [36-40]. In this study, we extend the application to detect all the base changes at a given codon of *K-RAS *gene simultaneously and established it as a simple and fast way to screen *K-RAS *gene. Our results indicated a strong association between *K-RAS *mutation in exon 2 and clinical outcome in terms of survival, lymph node involvement, and tumor size. Mutated exon 2 of *K-RAS*, thus represents a molecular lesion in the development of more aggressive disease. The effectiveness of cetuximab in colorectal cancer is strongly associated with *K-RAS *mutation status [12]. The method that we demonstrated in this report provides a simple, fast, and reliable way to identify *K-RAS *mutation for the purpose of clinical evaluation in colorectal cancer. Meanwhile, the principle of the method can also be used to detect *H-RAS *and *N-RAS *mutations, or to detect the mutation at a given codon in other genes in colorectal and other cancers

## Methods

### Samples

Resected primary colorectal cancers were obtained from 228 patients in Changhua Christian Hospital. All the cases were adenocarcinomas and were classified as "poorly-differentiated" to "well-differentiated". All patients were staged according to the 2002 American Joint Committee on Cancer staging system [41]. The tumor specimens were frozen immediately after surgical resection and stored in liquid nitrogen until DNA was extracted. The age of the patients ranged from 36–72 years with a mean of 49 years. This study was approved by the Institute Review Board of Changhua Christian Hospital.

### DNA extraction, PCR and direct sequencing of the *K-RAS *gene

DNA extraction was performed as previously described [33]. Three pairs of primers were used to amplify the exon-intron junctions and coding regions of exons 2, 3, and 4 of the *K-RAS *gene (see Additional file 1). The PCR was performed with a denaturing step at 94°C for 5 min, then 30 sec at 94°C, 30 sec at 56°C, and 1 min at 72°C for 35 cycles, followed by a final 5 min at 72°C. The PCR products were visualized on a 2.5% agarose gel. These PCR products were then subjected to direct sequencing using the same primers, and all mutations were confirmed by sequences originating from both the upstream and downstream primers. Direct sequencing was performed on a Beckman Coulter CEQ 8000 Series Genetic Analysis System (Beckman Coulter Inc., Fullerton, CA, USA) according to manufacturer instructions.

### Multiplex PCR and primer extension analysis of mutations in *K-RAS *codons 12, 13, 61 and 146

Multiplex PCR was used to amplify exons 2, 3 and 4 of *K-RAS *gene in a single tube. The primers used for the multiplex PCR were identical to those used for the direct sequencing analysis. After multiplex PCR amplification, the PCR products were purified to remove the remaining primers and unincorporated deoxynucleotide triphosphates, using the PCR-M™ Clean Up System (Viogene-biotek Co., Sunnyvale, CA, USA) or a simple treatment with exonuclease I and shrimp alkaline phosphatase. After removing the primers, the products were subjected to primer extension analysis. The primers ranged from 20–65 bases, and most probed the sense strand (see Additional file 2). The exception was when there were adjacent mutations; in this case both sense and antisense strand primers were used. Degenerate bases were used in the primers to identify adjacent mutations. Various concentrations of probe for either codon 12, 13, 61 or 146, were added to the tube containing purified PCR products, as well as 4 μl of SNPStart Master Mix (Beckman Coulter Inc., Fullerton, CA, USA) containing Taq DNA polymerase and fluorescently labelled ddNTPs. Each 10-μl mixture was subjected to 30 single-base extension cycles consisting of a denaturation step at 90°C for 10 sec, and primer annealing and extension at 55°C for 20 sec. After cycle extension, unincorporated fluorescent ddNTPs were incubated with 0.25 μl of shrimp alkaline phosphatase (SAP) (United States Biochemical Co., Cleveland, USA), 1.3 μl SAP buffer, and 1.45 μl ddH_2_O at 37°C for 30 min, followed by enzyme deactivation at 65°C for 15 min.

The primer extension reaction products were resolved by automated capillary electrophoresis on a capillary electrophoresis platform. Briefly, 38.75 μl of sample loading solution and 0.25 μl of Size Standard 80 (Beckman Coulter Inc., Fullerton, CA, USA) were added to 1 μl of primer extension products. The fluorescently tagged extended products in the mixture were electrophoretically separated across a 36-cm capillary containing POP-4 for 17 min and analyzed using GeneScan application software (Beckman Coulter Inc., Fullerton, CA, USA)

### Statistics

Comparison between clinicopathological features and status of *K-RAS *mutation in colorectal cancers were analyzed by Chi-square test or Fisher's exact test. Independent prognostic factors were analyzed by the Cox proportional harzards regression model. Variables in the model included tumor size, histologic grade, and lymph node metastasis. A P value of less than 0.05 was considered statistically significant.

## Competing interests

The authors declare that they have no competing interests.

## Authors' contributions

YSC performed the experiments and drafted the manuscript. KTY helped to design the study. TJC and NCH participated in the statistical analysis. CC helped to design the study. HCL participated in the coordination of the study. JGC design the study. All authors read and approved the final manuscript.

## Pre-publication history

The pre-publication history for this paper can be accessed here:

http://www.biomedcentral.com/1471-2407/9/179/prepub

## Supplementary Material

Additional file 1 **Primers used to amplify exon-intron junctions and coding regions of exons 2, 3, and 4 of the *K-RAS *gene**. This table includes the primers used in the multiplex PCR of *K-RAS*. Click here for file

Additional file 2 **The sequences of primers used for detection of hotspot mutations of *K-RAS***. This table lists the primers used in the mutation analysis of *K-RAS *by the method of primer extension. Click here for file
