# Supplementary material for: Fast simultaneous detection of K-RAS mutations in colorectal cancer
Source: BMC Cancer. 2009 Jun 11;9:179. doi: 10.1186/1471-2407-9-179 (PMC2702390; doi:10.1186/1471-2407-9-179)
Supplement: Additional file 1 — Primers used to amplify exon-intron junctions and coding regions of exons 2, 3, and 4 of the K-RAS gene. This table includes the primers used in the multiplex PCR of K-RAS. [file 1471-2407-9-179-S1.doc]

**Additional file 1**. Primers used to amplify exon-intron junctions and coding regions of exons 2, 3, and 4 of the K-RAS gene

| exon | codon | primer sequence |
| --- | --- | --- |
| 2 | 1 to 37 | 5’-ACACGTCTGCAGTCAACTGG-3’ |
|  |  | 5’-TAACTTGAAACCCAAGGTAC-3’ |
| 3 | 38 to 97 | 5’-GCACTG­TAATAATCCAGACT-3’ |
|  |  | 5’-CATGGCATTAGCAAAGACTC-3’ |
| 4 | 98-150 | 5’-GACAAAAGTTGTGGACAGGT-3’ |
|  |  | 5’-TAGCATAATTGAGAGAAAAACTG-3’ |
