# Supplementary material for: Fast simultaneous detection of K-RAS mutations in colorectal cancer
Source: BMC Cancer. 2009 Jun 11;9:179. doi: 10.1186/1471-2407-9-179 (PMC2702390; doi:10.1186/1471-2407-9-179)
Supplement: Additional file 2 — The sequences of primers used for detection of hotspot mutations of K-RAS. This table lists the primers used in the mutation analysis of K-RAS by the method of primer extension. [file 1471-2407-9-179-S2.doc]

**Additional file 2**. The sequences of primers used for detection of hotspot mutations of *K-RAS*.

| **codon12 first base** | **5'-AACTTGTGGTAGTTGGAGCT3'** |
| --- | --- |
| **codon12 second base** | **5'-ACTGAATATAAACTTGTGGTAGTTGGAGCTG3'** |
|  | **5'-ACTGAATATAAACTTGTGGTAGTTGGAGCTN3'** |
|  | **5'-GTATCGTCAAGGCACTCTTGCCTACGCCA3'** |
| **codon13 first base** | **5'-TGAAAATGACTGAATATAAACTTGTGGTAGTTGGAGCTGGT3'** |
| **codon13 second base** | **5'-GCCTGCTGAAAATGACTGAATATAAACTTGTGGTAGTTGGAGCTGGTG3'** |
|  | **5'-GCCTGCTGAAAATGACTGAATATAAACTTGTGGTAGTTGGAGCTGGTN3'** |
|  | **5'-CACAAAATGATTCTGAATTAGCTGTATCGTCAAGGCACTCTTGCCTACG3'** |
| **codon61 first base** | **5'-GCAAGTAGTAATTGATGGAGAAACCTGTCTCTTGGATATTCTCGACACAGCAGGT-3'** |
| **codon61 second base** | **5'-GGAAGCAAGTAGTAATTGATGGAGAAACCTGTCTCTTGGATATTCTCGACACAGCAGGTC-3'** |
| **codon61 thrid base** | **5'-(T)45ATTCTCGACACAGCAGGTCA-3'** |
| **codon146 first base** | **5'- ATGGAATTCCTTTTATTGAAACATCA-3'** |
| **codon146 second base** | **5'-(T)10ATGGAATTCCTTTTATTGAAACATCAG-3'** |
